# Medical Opinion and Movement

**Published:** 1907-08-24

**Authors:** 


### Medical Opinion and Movement.

On the Continent there is a very general fear of the evils which may result from the use of ordinary water for drinking purposes. In consequence there is an enormous consumption of so-called "table waters," aerated or otherwise. People are ready to pay for what they could otherwise obtain free, in the sublime belief that they are securing a drinking water free from any germs of disease or other impurity. Anyone who reads the report of M. Bruhat on his analysis of a large number of table waters, including all those most in vogue, will suffer a rude awakening. He finds that the majority of these waters fall far below the standard of purity which may reasonably be expected of them, and that ordinary water which the public rejects as suspicious contains fewer micro-organisms than these table waters, sold at relatively high prices, and consumed by the public on account of their faith in the purity of them. M. Bruhat was so surprised at his own findings that he repeated his analysis on a larger scale. He suggests as remedies that the public should be made aware of the actual condition of things, and that the authorities should exercise a more rigorous control over the industry.

Flushing the system with large quantities of fluid is now regarded as an important factor in the treatment of conditions of general peritonitis. Dr. Murphy, of Chicago, and Dr. Le Conte, of Philadelphia, attribute their success in the treatment of these conditions largely to their method of injecting large quantities of fluid into the rectum. Mr. B. G. A. Moynihan, of Leeds, following their method, is equally impressed with the efficacy of the treatment. Using the method in 19 consecutive cases of general peritonitis due to appendicitis, he has had only two deaths, and he believes the recovery in many cases due to the continuous administration by the rectum of saline solution over a period of two to four days after operation. In a recent paper he gives the details of technique, by which a continual flow may be secured. The rectal tube he uses is similar to the ordinary vaginal flushing tube with several apertures, and this communicates by rubber tubing with a supply flask arranged on the "wash-bottle" principle to give a continuous flow. It is provided with a thermometer, and the temperature of the saline is kept at about 100° F. by immersion in a bath of hot water provided with a spirit lamp. It is, of course, necessary that there should be an intelligent nurse in constant attendance. The flow should be at the rate of about 1 pint per hour, and as much as 16 pints may be given in the course of 24 hours. The effect upon the patient Mr. Moynihan describes as remarkable. Instead of the sunken features, parched mouth and clammy skin, the patient soon begins to exhibit a clean and ruddy skin, bright eyes, a moist tongue, and an aspect of comfort and contentment.

The London School of Medicine for Women is to be congratulated on the successful stand it has made in conjunction with the British Medical Association in support of the principle that there should be no difference in the rate of remuneration for official appointments for the two sexes. In several cases attempts have been made by Boards of Guardians to effect an economy by advertising for female medical officers at a lower salary than has been given to their male predecessors. The recent dispute at Halifax was a case in point. The Halifax Board of Guardians advertised for a resident lady medical officer at the Halifax Union Hospital at a salary of £100 per annum. Previously the appointment had been held by a male doctor at £140. The services of a lady doctor having been obtained at the reduced salary, the matter was taken up by the London School of Medicine for Women and the local division of the British Medical Association, and the lady appointed resigned the post before entering upon her duties. A second lady doctor, who accepted the appointment in ignorance of the dispute, also resigned on being made aware of the facts of the case. As a result of this firm attitude assumed by the profession, the Board of Guardians has sought to effect a compromise, and the dispute has been amicably settled. It is most important that in such cases women should steadfastly refuse to undersell their medical brethren, and should maintain the principle that there should be no difference in remuneration on the ground of sex. By so doing they will not only serve their own interests, but also those of the profession at large.

The tuberculin reaction as a means of diagnosis is already well recognised. It has been shown recently that instead of injecting the tuberculin hypodermically, a local reaction may be obtained by introducing it into a small scarified area of skin. In 48 hours the area becomes red and œdematous, and frequently a sort of papule appears, which dries up and disappears again in the course of a week. Wolff has obtained a similar reaction by introducing tuberculin into the eyes of bovines. Following these results M. Calmette has carried out a series of experiments on the human subject. He has treated 25 individuals, of whom 16 were tuberculous and nine affected with other diseases not tuberculous. He instilled one drop of a 1 per cent. aqueous solution of tuberculin into the eye of each patient. In the case of all the tuberculous patients the palpebral conjunctiva showed congestion and œdema 3 to 5 hours afterwards, with a fibrinous secretion and lachrymation. These signs subsided in the course of 24 to 36 hours, and caused only slight inconvenience, but no pain. In the other cases at the most only a slight redness appeared 2 or 3 hours afterwards, which rapidly disappeared. M. Calmette designates this the "ophthalmo-reaction to tuberculin." If further investigation proves it to be as harmless and as certain a diagnostic sign as these experiments suggest, it may prove of value to the clinician. The profession on this side shows, however, less temerity in adopting methods of diagnosis or treatment of uncertain value, and further confirmation will doubtless be awaited before medical men will ask their patients to submit to an instillation of tuberculin into their eyes.

